# A new model predicts hepatocellular carcinoma in patients with HBV-related decompensated liver cirrhosis and long-term antiviral therapy: a prospective study

**DOI:** 10.7717/peerj.15014

**Published:** 2023-03-24

**Authors:** Hao-dan Mao, Shu-qin Zheng, Su-hua Yang, Ze-yu Huang, Yuan Xue, Min Zhou

**Affiliations:** 1 Institute of Hepatology, Changzhou Third People’s Hospital, Changzhou, Jiangsu, China; 2 Department of Infectious Diseases, Changzhou Third People’s Hospital, Changzhou Medical Center, Nanjing Medical University, Changzhou, Jiangsu, China

**Keywords:** Hepatocellular carcinoma, Risk score, Liver cirrhosis, Decompensated cirrhosis, Prediction

## Abstract

**Background:**

We aimed to evaluate the prediction values of non-invasive models for hepatocellular carcinoma (HCC) development in patients with HBV-related liver cirrhosis (LC) and long-term NA treatment.

**Methods:**

Patients with compensated or decompensated cirrhosis (DC), who achieved long-term virological response, were enrolled. DC and its stages were defined by the complications including ascites, encephalopathy, variceal bleeding, or renal failure. Prediction accuracy of several risk scores, including ALBI, CAMD, PAGE-B, mPAGE-B and aMAP, was compared.

**Results:**

The median follow-up duration was 37 (28–66) months. Among the 229 patients, 9 (9.57%) patients in the compensated LC group and 39 (28.89%) patients in the DC group developed HCC. The incidence of HCC was higher in the DC group ( $\cal X$^2^ = 12.478, *P* < 0.01). The AUROC of ALBI, aMAP, CAMD, PAGE-B and mPAGE-B scores were 0.512, 0.667, 0.638, 0.663, 0.679, respectively. There was no significant difference in AUROC between CAMD, aMAP, PAGE-B and mPAGE-B (all *P* > 0.05). Univariable analysis showed that age, DC status and platelet were associated with HCC development, and multivariable analysis showed that age and DC status (both *P* < 0.01) were independent risk factors for HCC development, then Model (Age_DC) was developed and its AUROC was 0.718. Another model, Model (Age_DC_PLT_TBil) consisting of age, DC stage, PLT, TBil was also developed, and its AUROC was larger than that of Model (Age_DC) (0.760 *vs*. 0.718). Moreover, AUROC of Model (Age_DC_PLT_TBil) was larger than the other five models (all *P* < 0.05). With an optimal cut-off value of 0.236, Model (Age_DC_PLT_TBil) achieved 70.83% sensitivity, 76.24% specificity.

**Conclusion:**

There is a lack of non-invasive risk scores for HCC development in HBV-related DC, and a new model consisting of age, DC stage, PLT, TBil may be an alternative.

## Introduction

Liver cancer is one of the most common cancers worldwide, of which over 90% of the cases were hepatocellular carcinoma (HCC) (Su et al., 2022). The outcomes vary based on the severity of the underlying chronic liver disease and the tumor stage. HCC diagnosed at an intermediate or advanced stage, is related to a poor prognosis (Foerster et al., 2022).

Despite long-term antiviral therapy, the risk of HCC remains high among patients with chronic HBV infection (Kim et al., 2020; Zhu et al., 2020). It is striking that HCC developed in 31.8% of the patients with decompensated cirrhosis (DC) during a 5-year follow-up (Zhu et al., 2020). Considering that the incidence of HCC supports that increasing and early diagnosis improves the patients’ prognosis, HCC surveillance is recommended, particularly in patients with liver cirrhosis (LC).

At the present time, several non-invasive models for HCC prediction have been reported, including risk estimation for HCC in chronic hepatitis B (CHB) score (REACH-B) (Yang et al., 2011), cirrhosis, age, male, and diabetes score (CAMD) (Hsu et al., 2018), platelets, age and gender score (PAGE-B) (Papatheodoridis et al., 2016), the modified PAGE-B score (mPAGE-B) (Kim et al., 2018), age, male, albumin-bilirubin, and platelets (aMAP) (Fan et al., 2020), albumin-bilirubin (ALBI) score (Casadei Gardini et al., 2019). PAGE-B, for which AUROC was higher than that of REACH-B, can easily identify high-risk cases of HCC in patients treated with nucleot(s)ide analogs (NAs) (Kirino et al., 2020). For patients with compensated LC, aMAP showed better predictive performance than CAMD, mPAGE-B and PAGE-B, according to the Harrell’s c-index (Gui et al., 2021).

It should be noted that patients with DC were excluded from the above studies (Hsu et al., 2018; Papatheodoridis et al., 2016; Kim et al., 2018; Fan et al., 2020; Kirino et al., 2020; Lee et al., 2019). To date, there is limited information about the non-invasive models for HCC surveillance in patients with DC. Herein, we investigated the predictive value of the non-invasive prediction models in patients with HBV-related LC and long-term NA treatment. Moreover, a new model consisting of age, DC stage, platelet, and total bilirubin (TBil), was developed and it can identify patients with a high risk of HCC.

## Methods

### Patients and primary endpoint

From May 2010 to December 2021, 307 patients with LC admitted to the Third People’s hospital of Changzhou, were recruited, and were prospectively followed up. HBV-related compensated LC and DC were diagnosed according to Chinese guidelines for prevention and treatment of CHB (2019 version) (Jia et al., 2020). Patients with nodules in the hepatic parenchyma found in histological or ultrasonographic examination, or gastroesophageal varices detected by endoscopic evaluation, were diagnosed with compensated LC. DC and its stages were defined by the complications including ascites, encephalopathy, variceal bleeding, or hepatorenal syndrome (D’Amico, Garcia-Tsao & Pagliaro, 2006; D’Amico et al., 2018). HCC was diagnosed according to the imaging techniques, alpha-fetoprotein and/or histological findings (Health Commission of the People’s Republic of China, 2020). Patients co-infected with other hepatitis virus or suffered from malignant tumor, were excluded. Patients who had positive HBV DNA at the end of follow-up, developed HCC within 6 months during follow-up, or lost to follow-up were also excluded.

All the patients received NAs treatment, including Lamivudine, Adefovir, Telbivudine, Entecavir or Tenofovir at admission, and routinely underwent clinical examination, laboratory tests and ultrasonography every 3 to 6 months. Demographic and clinical data, including age, sex, alanine transaminase (ALT), aspartate transaminase (AST), total bilirubin (TBil), gamma-glutamyl transpeptidase (GGT), albumin, international standard ratio (INR), HBV serologic markers, serum HBV DNA, blood cell count, and complications were collected at the first time of admission. The endpoints of the study were HCC development or death.

The study was non-interventional and not harmful to the patients, and written consents were obtained from all the participants. The protocol was approved by the Ethics Committee of the Third People’s Hospital of Changzhou according to the Declaration of Helsinki, 2013 (No. CZSY2018-0601).

### Score systems

Risk scores including CAMD (Hsu et al., 2018), PAGE-B (Papatheodoridis et al., 2016), mPAGE-B (Kim et al., 2018), aMAP (Fan et al., 2020), ALBI score (Casadei Gardini et al., 2019) were calculated as described before.

### Statistical analysis

All data were analyzed using SPSS version 25.0 (Armonk, NY, USA). Continuous variables were presented as median (interquartile range, IQR), and were analyzed using the Mann-Whitney U tests. Categorical values were presented as frequencies, and were compared using the chi-square test. Correlation analysis was evaluated using the Spearman correlation test. Independent risk factors for HCC development were identified using univariate and multivariate logistic regression analysis. Accuracy of the scoring systems was compared according to the area under the receiver operating characteristic curve (AUROC), which was calculated using MedCalc version 15.2.2 software for Windows (Medcalc Software, Mariakerke, Belgium). The cutoff value was identified using MedCalc software, and then Kaplan-Meier analysis was performed using GraphPad Prism version 5.0 for Windows (GraphPad Software, San Diego, CA, USA). *P* value < 0.05 was considered statistically significant.

## Results

### Characteristics of patients

**Figure 1:**
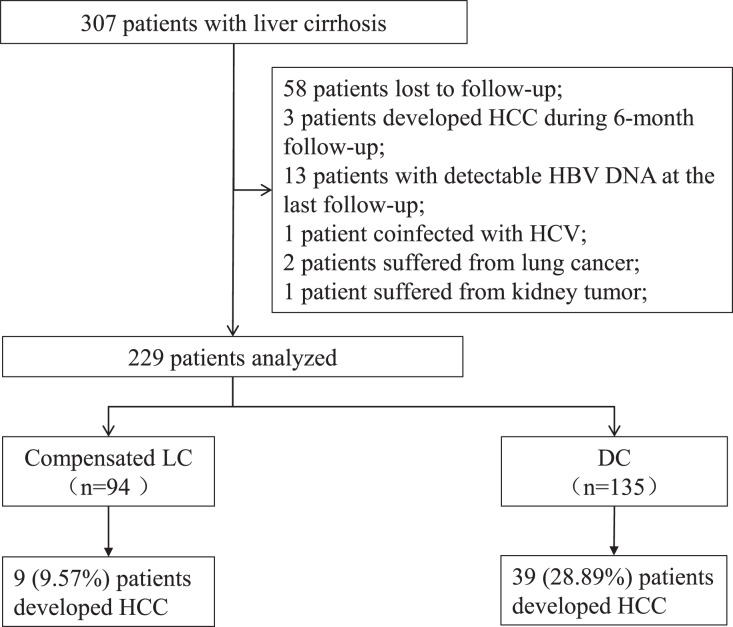
Screening of patients with liver cirrhosis. LC, liver cirrhosis; DC, decompensated cirrhosis; HCC, hepatocellular carcinoma.

Among the 307 patients, 78 patients were excluded, including 58 patients lost to follow-up, three patients developed HCC during 6 months of follow-up, 13 patients had positive HBV DNA at the last visit, one patient coinfected with hepatitis C virus, two patients suffered from lung cancer, and one patient suffered from kidney tumor. Data from 229 patients were analyzed, including 94 individuals with compensated LC and 135 individuals with DC (Fig. 1). Of the 135 patients with DC, 12 patients had hepatic encephalopathy, six patients had variceal bleeding, 121 patients had ascites, and one patient had renal failure. A total of 28 patients were stratified as late decompensated state based on the refractory ascites, recurrent encephalopathy, and renal failure.

The median follow-up duration was 37 (28–66) months. A total of 9 (9.57%) patients in the compensated LC group and 39 (28.89%) patients in the DC group developed HCC. The incidence of HCC was higher in the DC group ( $\cal X$^2^ = 12.478, *P* < 0.01).

The characteristics of the 48 patients who developed HCC are shown in Table 1. Patients who developed HCC were older than those without HCC (*P* < 0.01). CAMD, PAGE-B, mPAGE-B and aMAP scores were higher, while the platelet count was lower in patients with HCC (*P* < 0.01).

**Table 1:** Characteristics of patients with HBV-related liver cirrhosis.

Variables	Patients did not developed HCC (*n* = 181)	Patients developed HCC (*n* = 48)	*Z* or $\cal X$^2^	*P* value
Age (years)	50.0 (45.0–60.0)	57.5 (51.5–64.0)	−3.850	<0.01
Male, *n* (%)	119 (65.8)	34 (70.8)	0.443	0.51
DC, *n* (%)	96 (53.0)	39 (81.3)	12.478	<0.01
Diabetes, *n* (%)	52 (28.7)	17 (35.4)	0.806	0.37
ALT, U/L	33.0 (21.0–65.0)	34.0 (23.0–56.5)	−0.168	0.87
AST, U/L	36.0 (25.0–54.0)	42.5 (31.3–73.0)	−1.308	0.19
GGT, U/L	50.0 (25.5–79.0)	64.5 (26.5–107.8)	−1.578	0.11
TBil, µmol/L	22.5 (15.4–34.7)	20.0 (14.6–31.8)	0.566	0.57
Albumin, g/L	39.1 (32.8–43.3)	38.0 (31.0–42.2)	0.787	0.43
Creatinine, µmol/L	77.7 (68.4–92.0)	77.9 (66.6–92.6)	0.464	0.64
PLT, E+09/L	96.0 (56.5–141.0)	70.5 (53.5–101.5)	2.684	<0.01
Serum sodium, mmol/L	141.0 (139.4–142.2)	141.0 (139.4–142.3)	−0.117	0.91
INR	1.1 (1.1–1.3)	1.2 (1.1–1.3)	−0.826	0.41
ALBI	−2.5 (−2.8 to −1.8)	−2.3 (−2.8 to −1.6)	−0.533	0.59
CAMD	14.0 (13.0–17.0)	16.0 (14.3–18.0)	−4.061	<0.01
PAGE_B	16.0 (14.0–19.5)	19.0 (17.3–21.0)	−3.939	<0.01
mPAGE_B	12.0 (11.0–15.0)	15.0 (13.3–17.0)	−4.412	<0.01
aMAP	60.0 (54.5–65.8)	66.5 (62.6–69.0)	−4.399	<0.01
Model (Age_ DC_PLT_TBil)	0.1 (0.1–0.2)	0.3 (0.2–0.4)	−5.538	<0.01
MELD score	10.0 (8.0–13.0)	9.0 (8.0–12.8)	0.244	0.81
Duration of follow-up, months	38.0 (30.5–66.5)	30.0 (19.0–60.0)	2.189	0.03

**Notes:**

Comparison was conducted by a Mann-Whitney U test (median and IQR) for continuous variables, and Chi-square test for categorial values.

HCC, hepatocellular carcinoma; DC, decompensated cirrhosis; ALT, alanine aminotransferase; AST, aspartate aminotransferase; GGT, gamma-glutamyl transpeptidase; TBil, total bilirubin; PLT, Platelet; INR, international normalized ratio; ALBI, albumin-bilirubin score; CAMD, cirrhosis, age, male, and diabetes score; PAGE-B, platelets, age and gender score; mPAGE-B, the modified PAGE-B score; aMAP, age, male, albumin-bilirubin, and platelets score; MELD, model for end-stage liver disease.

### Independent risk factors for HCC development

As shown in Table 2, the univariate logistic analysis showed that age, DC status and platelet were associated with HCC development. Multivariable analysis showed that age and DC status were independent risk factors for HCC development (both *P* < 0.01). Then a prediction models were developed: Model (Age_DC) (y = 1) = ${{\rm exp}\,\left( { -\ 3.658\ +\ 0.049\ *\ {\rm Age}\ -\ 1.107\ *\ {\rm DC}\left( 1 \right)} \right)} \over {1\ +\ {\rm exp}\,\left( { -\ 3.658 \,+\, 0.049\ *\ {\rm Age}\ -\ 1.107\ *\ {\rm DC}\left( 1 \right)} \right)}$. Considering that TBil and PLT were important indexes of several non-invasive models, another model incorporating TBil and PLT into Model (Age_DC), Model (Age_DC_PLT_TBil) (y = 1) = ${{\rm exp}\,\left( { -\ 2.406\ +\ 0.051\ *\ {\rm Age}\ -\ 1.090\ *\ {\rm DC}\left( 1 \right)\ -\ 0.022\ *\ {\rm TBil}\ -\ 0.008\ *\ {\rm PLT}} \right)} \over {1\ +\ {\rm exp}\,\left( { -\ 2.406\ +\ 0.051\ *\ {\rm Age}\ -\ 1.090{\rm DC}\left( 1 \right)\ -\ 0.022{\rm TBil}\ -\ 0.008\ *\ {\rm PLT}} \right)}$, was developed. The AUROC of the two models were compared, and Model (Age_DC_PLT_TBil) was more potent than Model (Age_DC) (AUROC: 0.760 and 0.718). For patients with compensated LC, AUROC of Model (Age_DC_PLT_TBil) was larger than Model (Age_DC) (AUROC: 0.778 and 0.684). For patients with DC, AUROC of Model (Age_DC_PLT_TBil) was also larger than Model (Age_DC) (AUROC: 0.691 and 0.629). Patients who developed HCC had significantly higher Model (Age_DC_PLT_TBil) scores than those patients who did not develop HCC (*Z* = 5.538, *P* < 0.01).

**Table 2:** Risk factors for HCC in patients with liver cirrhosis.

Baseline variables	Univariate				Multivariate		
	Odds ratio	95% CI	*P*		Odds ratio	95% CI	*P*
Age	1.062	[1.027–1.098]	<0.01		1.050	[1.015–1.086]	<0.01
Male	1.265	[0.632–2.533]	0.51				
DC	0.261	[0.119–0.569]	<0.01		0.331	[0.148–0.739]	<0.01
ALT	0.998	[0.994–1.001]	0.24				
AST	0.999	[0.995–1.002]	0.44				
Albumin	0.979	[0.935–1.026]	0.38				
TBil	0.998	[0.980–1.017]	0.87				
INR	1.540	[0.408–5.812]	0.52				
Creatinine	0.997	[0.985–1.009]	0.61				
Serum sodium	0.994	[0.874–1.130]	0.93				
PLT	0.988	[0.979–0.997]	0.01				
WBC	0.922	[0.787–1.081]	0.32				
Diabetes	0.735	[0.375–1.441]	0.37				

**Note:**

HCC, hepatocellular carcinoma; DC, decompensated cirrhosis; ALT, alanine aminotransferase; AST, aspartate aminotransferase; TBil, total bilirubin; INR, international normalized ratio; PLT, Platelet; WBC, white blood cell.

### Comparison of ALBI, aMAP, CAMD, PAGE-B, mPAGE-B and Model (Age_DC_PLT_TBil)

**Figure 2:**
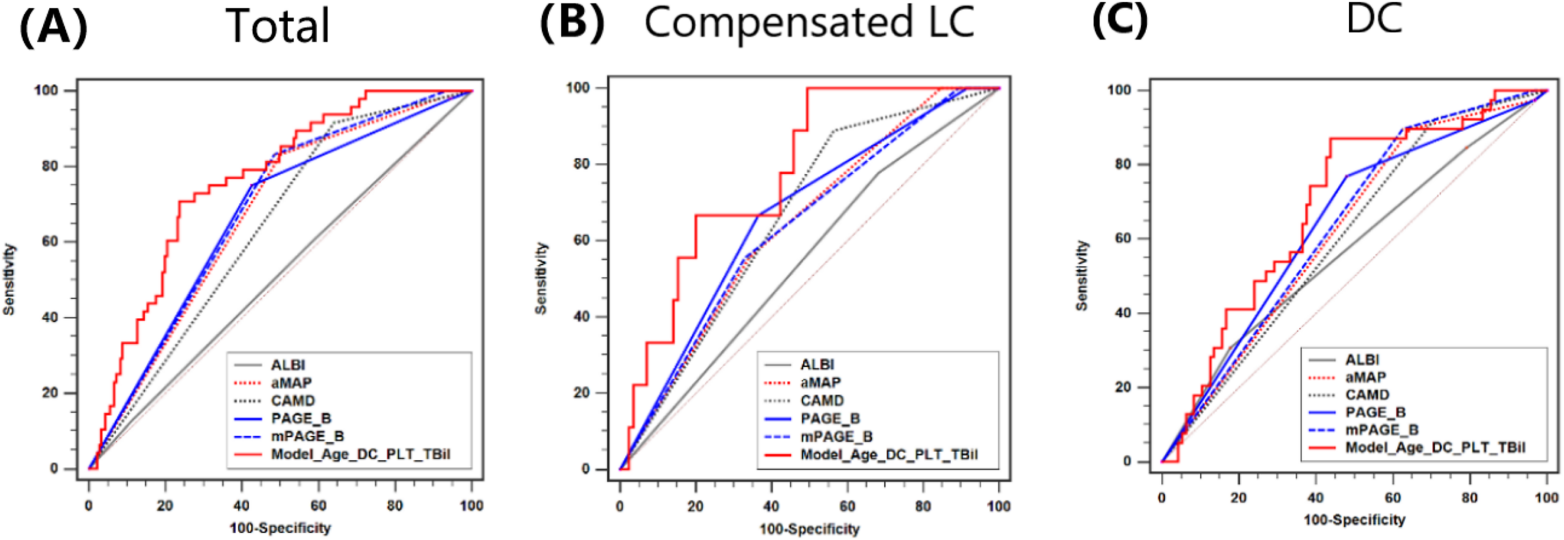
Comparison of AUROC between ALBI, CAMD, aMAP, PAGE-B and mPAGE-B and Model (Age_DC_PLT_TBil). (A) Total population; (B) compensated liver cirrhosis; (C) decompensated cirrhosis.

As shown in Fig. 2, the AUROC of ALBI, aMAP, CAMD, PAGE-B, mPAGE-B and Model (Age_DC_PLT_TBil) scores were 0.512, 0.667, 0.638, 0.663, 0.679 and 0.760, respectively. There was no significant difference in AUROC between CAMD, aMAP, PAGE-B and mPAGE-B (all *P* > 0.05). Moreover, AUROC of Model (Age_DC_PLT_TBil) was larger than the other five models (all *P* < 0.05). Moreover, the AUROC of Child-Pugh score in predicting HCC development was just 0.529, which was comparable with ALBI.

For patients with compensated LC, AUROC of ALBI, aMAP, CAMD, PAGE-B, mPAGE-B and Model (Age_DC_PLT_TBil) were 0.548, 0.641, 0.662, 0.665, 0.637 and 0.778, respectively. Model (Age_DC_PLT_TBil) had larger AUROC than mPAGE-B (*P* < 0.01) and ALBI (*P* = 0.02).

For patients with DC, AUROC of ALBI, aMAP, CAMD, PAGE-B, mPAGE-B and Model (Age_DC_PLT_TBil) were 0.574, 0.623, 0.607, 0.642, 0.638 and 0.691, respectively. AUROC of Model (Age_DC_PLT_TBil) seemed to be larger than those of ALBI (*P* = 0.05) and CAMD (*P* = 0.07).

### Correlation between ALBI, aMAP, CAMD, PAGE-B, mPAGE-B and Model (Age_DC_PLT_TBil)

**Figure 3:**
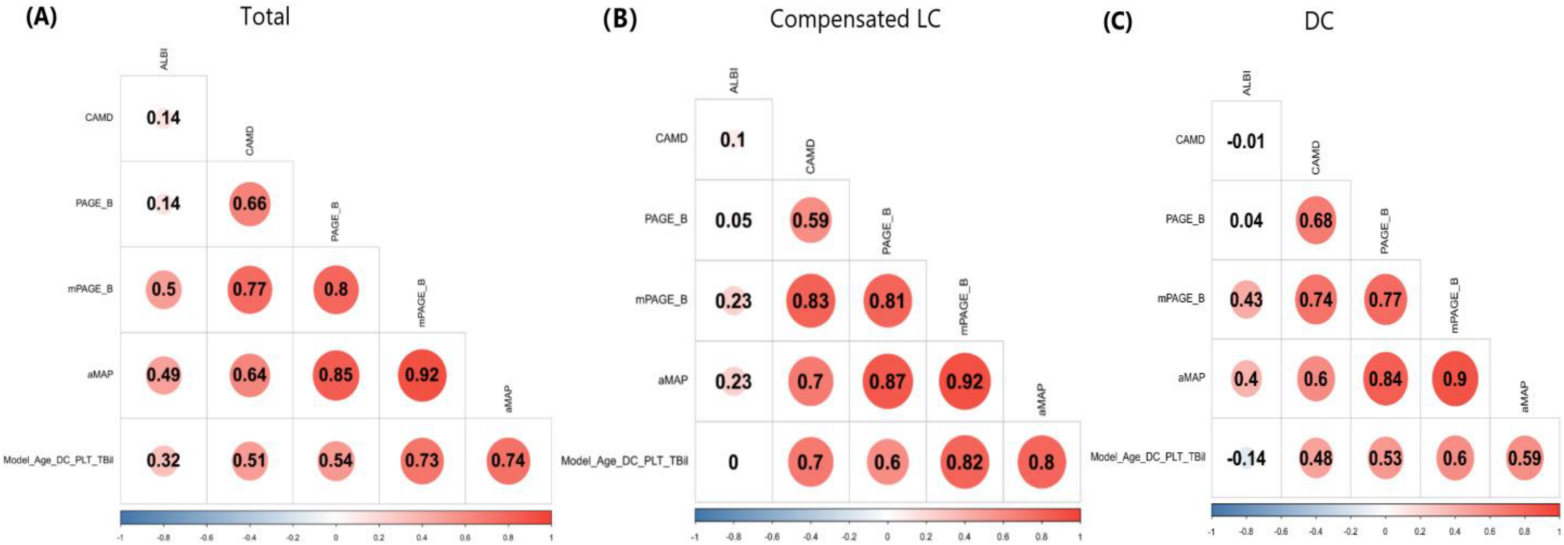
Correlation analysis between ALBI, CAMD, aMAP, PAGE-B and mPAGE-B and Model (Age_DC_PLT_TBil). (A) Total population; (B) compensated liver cirrhosis; (C) decompensated cirrhosis.

Model (Age_DC_PLT_TBil) positively correlated with CAMD, PAGE_B, mPAGE_B and aMAP (all *P* < 0.01), but not with ALBI (*P* = 0.99 and 0.10) in patients with compensated LC or DC. Moreover, for patients with compensated LC or DC, ALBI correlated with mPAGE_B and aMAP (all *P* < 0.05), but not with other three models (Fig. 3).

### Risk stratification for cumulative incidence of HCC

**Figure 4:**
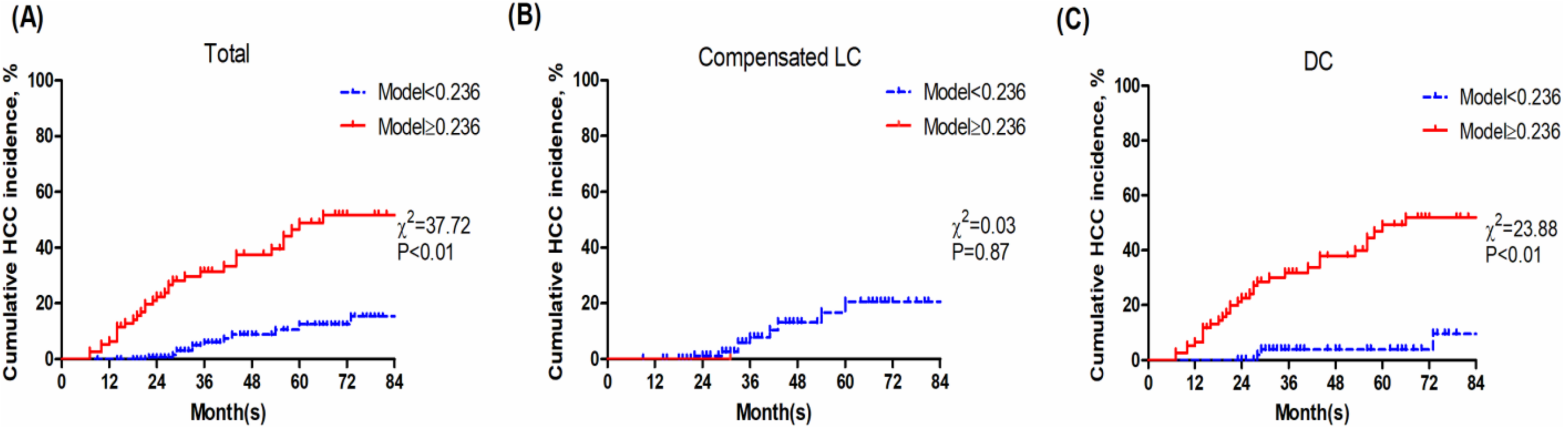
Kaplan-Meier analysis of Model (Age_DC_PLT_TBil). (A) Total population; (B) compensated liver cirrhosis; (C) decompensated cirrhosis.

With an optimal cut-off value of 0.236, Model (Age_DC_PLT_TBil) achieved 70.83% sensitivity, 76.24% specificity. Then the patients were divided into two groups: the low-risk group (Model (Age_DC_PLT_TBil) <0.236) and high-risk group (Model (Age_DC_PLT_TBil) ≥0.236). Patients with DC in high-risk group had a poor prognosis (*P* < 0.01) (Fig. 4).

## Discussion

Data from the present study showed that approximately one third (28.89%) patients in the DC group developed HCC during a median duration of 37-month follow-up. Although sustained virological response was achieved by NAs treatment, patients with DC remain at high risk of HCC (Zhu et al., 2020). Therefore, HCC surveillance should be paid more attention in clinical practice. Moreover, Child-Pugh score which is a common non-invasive scoring system in evaluating the severity and outcomes of LC, has a poor predictive accuracy according to the AUROC (0.529). To date, for patients with DC, these is no available models for HCC prediction (Lee et al., 2022; Yu et al., 2022).

Several non-invasive models have been investigated in patients with CHB or compensated LC (Hsu et al., 2018; Papatheodoridis et al., 2016; Kim et al., 2018; Fan et al., 2020; Kirino et al., 2020; Lee et al., 2019), or chronic hepatitis C (Casadei Gardini et al., 2019). The present study focuses on the predictive accuracy of these models in patients with DC. Comparison of AUROC shows that ALBI was not suitable for HCC prediction in HBV-related LC. In addition, aMAP, CAMD, PAGE-B and mPAGE-B did not seem to be potent yet (all AUROC <0.7). Based on a logistic regression analysis, Model (Age_DC_PLT_TBil) consisting of age, DC stage, platelets and TBil, was developed and it showed superiority in stratifying patients at high risk. Compared with aMAP (Gui et al., 2021) or transient elastography (Lee et al., 2021), this new model with potential clinical utility, is more easily to calculate, and it does not increase the economic burden to the patients. For patients at high-risk group, surveillance strategy including alpha-fetoprotein and imaging examinations are recommended to be performed routinely every 3 to 6 months (Sohn et al., 2022).

As shown in Fig. 4B, Model (Age_DC_PLT_TBil) shows non-optimal stratification accuracy for compensated LC. The main reason is that patients with Model (Age_DC_PLT_TBil) ≥0.236 are very few in the compensated LC group (just one patient), and the incidence of HCC is relatively lower. Normal PLT counts and TBil in patients with compensated LC and long-term antiviral therapy, result in lower Model (Age_DC_PLT_TBil) score. Moreover, baseline GGT seemed to be higher in patients who developed HCC, but the difference was not significant, then univariate logistic analysis showed that GGT was not related to HCC development. Recently, Huang et al. (2022) reported that GGT 6 months after initiating NAs strongly predicted HCC development in CHB patients, but not in LC patients. It would be interesting to analyze on-treatment GGT in patients with DC. Accumulated evidences are needed to validate its accuracy in studies with large sample-size.

It should be noted that HBV genotype, viral loading at baseline, mutation of HBV, as well as the kind of NAs, may influence the accuracy of Model (Age_DC_PLT_TBil). Recent data regarding the HBV genotype C2 in Korea, revealed that risk of HCC steadily persisted despite long-term antiviral treatment (Kim et al., 2020). It is still controversial that Tenofovir is superior to Entecavir in reducing the incidence of HCC (Kim et al., 2019; Ha et al., 2020; Chon et al., 2021; Lee et al., 2020). Moreover, HCC inhibition by different NAs remains largely unknown in patients with DC. Regardless of the kind of NAs, persistent HBV DNA positivity is speculated to be more harmful in patients undergoing long-term NAs treatment, especially for DC. Positive HBV DNA accompanying with abnormal TBil and decreased PLT counts due to hypersplenism, may present as high Model (Age_DC_PLT_TBil) score in patients with DC.

There are several limitations to the present study. First, in the present single-center study, the sample size is small and the follow-up duration is short, so multi-center study with large sample size is of importance for future research. Second, obesity and cirrhosis-associated immune dysfunction in patients with end-stage liver diseases may affect HCC development (Shin, Jun & Yi, 2022), and it should be interesting to evaluate the several models in patients with HBV-related LC and nonalcohol fatty liver diseases. Third, the family history of HCC, which has been confirmed to be a susceptible risk factor for HCC, was not collected in the present study.

In conclusion, it lacks of non-invasive risk scores for HCC development in HBV-related DC, and a new model consisting of Age, DC stage, PLT, TBil may be an alternative.

## Supplemental Information

10.7717/peerj.15014/supp-1 Supplemental Information 1 Raw data.

## Abbreviations

HCC: Hepatocellular carcinoma
LC: Liver cirrhosis
DC: Decompensated cirrhosis
CHB: Chronic hepatitis B
REACH-B: Risk estimation for HCC in chronic hepatitis B score
CAMD: Cirrhosis, age, male, and diabetes score
PAGE-B: Platelets, age and gender score
mPAGE-B: The modified PAGE-B score
aMAP: Age, male, albumin-bilirubin, and platelets score
ALBI: Albumin-bilirubin score
NA: Nucleot(s)ide analog
MELD: Model for end-stage liver disease
ALT: Alanine aminotransferase
AST: Aspartate aminotransferase
TBil: Total bilirubin
GGT: Gamma-glutamyl transpeptidase
INR: International normalized ratio
WBC: White blood cells
AUROC: Area under the receiver operating characteristic curve
Model (Age_DC_PLT_TBil): A model consisting of age, decompensated cirrhosis, platelet and total bilirubin

## Additional Information and Declarations

### Data Availability

The following information was supplied regarding data availability: The raw data is available in the Supplemental Files.
